# Resistance to Malaria by Enhanced Phagocytosis of Erythrocytes in LMP7-deficient Mice

**DOI:** 10.1371/journal.pone.0059633

**Published:** 2013-03-19

**Authors:** Xuefeng Duan, Takashi Imai, Bin Chou, Liping Tu, Kunisuke Himeno, Kazutomo Suzue, Makoto Hirai, Tomoyo Taniguchi, Hiroko Okada, Chikako Shimokawa, Hajime Hisaeda

**Affiliations:** 1 Department of Parasitology, Graduate School of Medical Sciences, Kyushu University, Fukuoka, Japan; 2 Department of Parasitology, Graduate School of Medicine, Gunma University, Maebashi, Japan; Osaka University, Japan

## Abstract

General cellular functions of proteasomes occur through protein degradation, whereas the specific function of immunoproteasomes is the optimization of antigen processing associated with MHC class I. We and others previously reported that deficiency in subunits of immunoproteasomes impaired the activation of antigen-specific CD8^+^ T cells, resulting in higher susceptibility to tumor and infections. We demonstrated that CD8^+^ T cells contributed to protection against malaria parasites. In this study, we evaluated the role of immunoproteasomes in the course of infection with rodent malaria parasites. Unexpectedly, *Plasmodium yoelii* infection of mice deficient in LMP7, a catalytic subunit of immunoproteasomes, showed lower parasite growth in the early phase of infection and lower lethality compared with control mice. The protective characteristics of LMP7-deficient mice were not associated with enhanced immune responses, as the mutant mice showed comparable or diminished activation of innate and acquired immunity. The remarkable difference was observed in erythrocytes instead of immune responses. Parasitized red blood cells (pRBCs) purified from LMP7-deficient mice were more susceptible to phagocytosis by macrophages compared with those from wild-type mice. The susceptibility of pRBC to phagocytosis appeared to correlate with deformity of the membrane structures that were only observed after infection. Our results suggest that RBCs of LMP7-deficient mice were more likely to deform in response to infection with malaria parasites, presumably resulting in higher susceptibility to phagocytosis and in the partial resistance to malaria.

## Introduction

The proteasome, a multicatalytic protease complex, is an essential component of the ATP-dependent proteolytic pathway that catalyzes the elimination of ubiquitinated proteins [1]. It is distributed in the nucleus and cytosol, where it can comprise 0.5 to 1.0% of total cellular protein [2]. The mammalian 26S proteasome is composed of a 20S proteolytic core consisting of two outer α rings, two inner β rings, and two additional 19S regulatory complexes. The 26S proteasome catalyzes the rapid degradation of proteins that are covalently linked to polyubiquitin chains. This pathway is highly regulated and selective, and in turn it regulates many important cellular processes such as transcriptional activation [3], cell-cycle progression [4], cell proliferation [5]–[7], differentiation [8], [9] and apoptosis [10], [11]. From the immunological point of view, proteasomal degradation of proteins is indispensable for antigen presentation associated with MHC class I, which activates CD8^+^ T cells [12].

Interferon (IFN)-γ is an immunomodulatory cytokine produced by activated CD4^+^ T cells, natural killer (NK) cells, and CD8^+^ T cells that enhances antigen presentation by activating proteasome subunits and regulators in addition to up-regulating the expression of MHC and TAP genes. IFN-γ alters proteasome activity by incorporation of three IFN-γ-inducible catalytic subunits, LMP2, LMP7, and MECL-1 to replace the constitutive catalytic subunits (Y/δ, X/MB1, and Z, respectively) in the 20S core particle during proteasome biogenesis [13]–[20]. These IFN-γ-induced immunoproteasomes are thought to be more favorable for antigen presentation than constitutive proteasomes because the subunits induced by IFN-γ contain chymotrypsin activity that cleaves hydrophobic, basic and branched chain residues instead of acidic residues [16], [21]–[23]. The importance of immunoproteasomes in MHC class I-associated antigen presentation has been proven using mice deficient for the IFN-γ-inducible subunits. Mice deficient in LMP7, a molecule responsible for major chymotrypsin activity, exhibited attenuated antigen presenting activity [24]. We previously showed that LMP7 plays a crucial role in inducing antigen-specific CD8^+^ T cells, and LMP7-deficient mice were more susceptible to tumors [25] and protozoan infection [26], [27], where CD8 T cells mainly function as effector cells.

Malaria remains a crucial threat to public health worldwide. It is well accepted that antibodies and CD4^+^ T cells play critical roles in protection against blood-stage malaria that can be acquired during natural or experimental infection [28]–[31]. In addition, innate immunity attributed to macrophages, NK cells and dendritic cells (DCs) is also important. Especially, phagocytosis exerted by macrophages residing in the reticuloendothelial system is crucial for the elimination of parasitized red blood cells (pRBCs). In contrast, the contribution of CD8^+^ T cells to protective immunity against blood-stage malaria is controversial. Although RBCs are exceptional cells that express no MHC class I molecules, CD8^+^ T cells are activated during blood-stage malaria [32], [33]. Furthermore, activation of CD8^+^ T cells is required for the development of experimental cerebral malaria [34]. We recently found that CD8 T cells are important for immunity against blood-stage malaria [35], leading us to hypothesize that LMP7-deficiency impairs resistance to infection with blood-stage malaria.

In this study, we observed that LMP7-deficient mice were partially resistant to infection with rodent malaria parasites, *Plasmodium yoelii*. We examined immune responses in LMP7-deficient mice in detail and found no explainable difference in innate and adaptive immunity including CD8 T cell responses. However, we found that pRBCs from LMP7-deficient mice were highly phagocytosed.

## Materials and Methods

### Ethics Statement

All experiments that involved mice were reviewed and approved by the Committee for Ethics on Animal Experiments in the Graduate School of Gunma University (approved number 12-031), and were conducted under the control of the Guidelines for Animal Experiments in the Graduate School of Medicine, Gunma University, and the Law (No. 105) and Notification (No. 6) of the Japanese Government.

### Mice and Parasites

C57BL/6 mice were purchased from Kyudo (Tosu, Japan). Immunoproteasome subunit LMP7-deficient (LMP7-deficient) mice on a C57BL/6 background were established [24] and provided by Dr. Fehling HJ (Ulm University, Germany). Age- and sex-matched groups of wild-type (WT) and LMP7-deficient mice were used for the experiments. Blood-stage parasites of *Plasmodium yoelii* 17XL(PyL) or 17XNL (PyNL) were obtained after fresh passage through a donor mouse 2–3 days after inoculation with a frozen stock. Mice were infected intraperitoneally with 25,000 parasitized pRBCs.

pRBCs were separated using a Percoll gradient after removal of leukocytes, as previously described [36]. Briefly, heparinized blood from malaria-infected mice was collected after heart puncture, and passed through a CF11 column to remove white blood cells. The eluent RBC solution was placed onto 58% (v/v) Percoll/PBS and centrifuged, and cells at the interphase or at the bottom were collected as schizont-rich pRBCs or schizont-free RBCs, respectively.

### Fluorescence-activated Cell Sorting (FACS) Analysis

The following antibodies (Abs) were obtained from eBioscience (San Diego, CA) and used to assess cell surface or intracellular expression of their respective antigens: allophycocyanin (APC)-conjugated anti-mouse CD3ε (145-2C11), APC-, FITC-, and PE-conjugated anti-mouse CD4 (GK1.5), PE-conjugated anti-mouse CD11b (M1/70), FITC-conjugated anti-mouse CD11c (N418), PE-Cy5-conjugated anti-mouse CD40 (1C10), FITC-conjugated anti-mouse CD69 (H1.2F3), APC-conjugated anti-mouse CD80 (16-10A1), APC-conjugated anti-mouse CD86 (GL1), PE-Cy5-conjugated anti-mouse I-A^b^ MHC class II (M5/144.15.2), and PE-conjugated anti-mouse IFN-γ (XMG1.2). Stained cells were analyzed using FACSCalibur flow cytometer (BD Bioscience, San Jose, CA), and the list data were analyzed using CellQuest Pro software (BD Biosciences).

### Quantitative real-time PCR

mRNA quantification of IFN-γ was performed with a real-time PCR system (Applied Biosystems, Foster City, CA), using SYBR Green I double-strand DNA binding dye. Total RNA extracted from 1×10^7^ splenocytes from an uninfected or infected mouse (5 days after infection) was reverse-transcribed followed by PCR. For IFN-γ, the sense and antisense primers were 5′-AGCGGCTGACTGAACTCAGATTGTAG-3′ and 5′-GTCACAGTTTTCAGCTGTATAGGG, respectively. Fluorescence data collected after each extension step were analyzed using an ABI Prism 7000 SDS software. The relative ratio of mRNA encoding IFN-γ in each sample was normalized to the relative quantity of β-actin.

### Phagocytosis of Macrophages

Peritoneal macrophages were collected from WT and LMP7-deficient mice 4 days after injection with 0.5 ml thioglycollate solution. RBCs (10^7^ cell/ml) were incubated with 10 µM carboxyfluorescein succinimidyl ester (CFSE) in PBS for 15 min at 37°C. CFSE staining was stopped by addition excess complete medium (fetal bovine serum-supplemented RPMI1640) and washing cells three times with complete medium. Macrophages (5×10^5^ or 4×10^5^ cells/well) were cultured with 5×10^6^ CFSE-labeled RBCs at a final volume of 200 µl for 1 h at 37°C. After co-culture, non-ingested RBCs were removed by hemolysis with NH_4_Cl lysing buffer. The remaining macrophages were washed twice with complete medium, and then stained with PE-conjugated anti-mouse CD11b Ab before flow cytometric analysis.

### Scanning Electron Microscopy (SEM)

The surface of RBCs was examined by SEM. RBCs from WT or LMP7-deficient mice were washed with PBS by repeated centrifugation at 1,000 × *g* for 10 min to remove contaminating cell debris and were then fixed with 1.5% glutaraldehyde. The specimen was then dehydrated in a series of acetone solutions and finally with amyl acetate. After critical point drying (JCPD2, JEOL), the RBC specimen was coated with gold-palladium for surface conductivity and examined by the scanning mode of the electron microscope (EMASID20 combined with JEM2000EX, JEOL) at 25 kV.

### Statistics

Differences between groups were analyzed for statistical significance with unpaired Student *t*-tests. For survival curves, Kaplan-Meier plots were performed. All of these were performed using Excel software. Probabilities below 0.05 were considered statistically significant.

## Results

### LMP7-deficient Mice are Partially Resistant to Infection with Malaria Parasites

**Figure 1:**
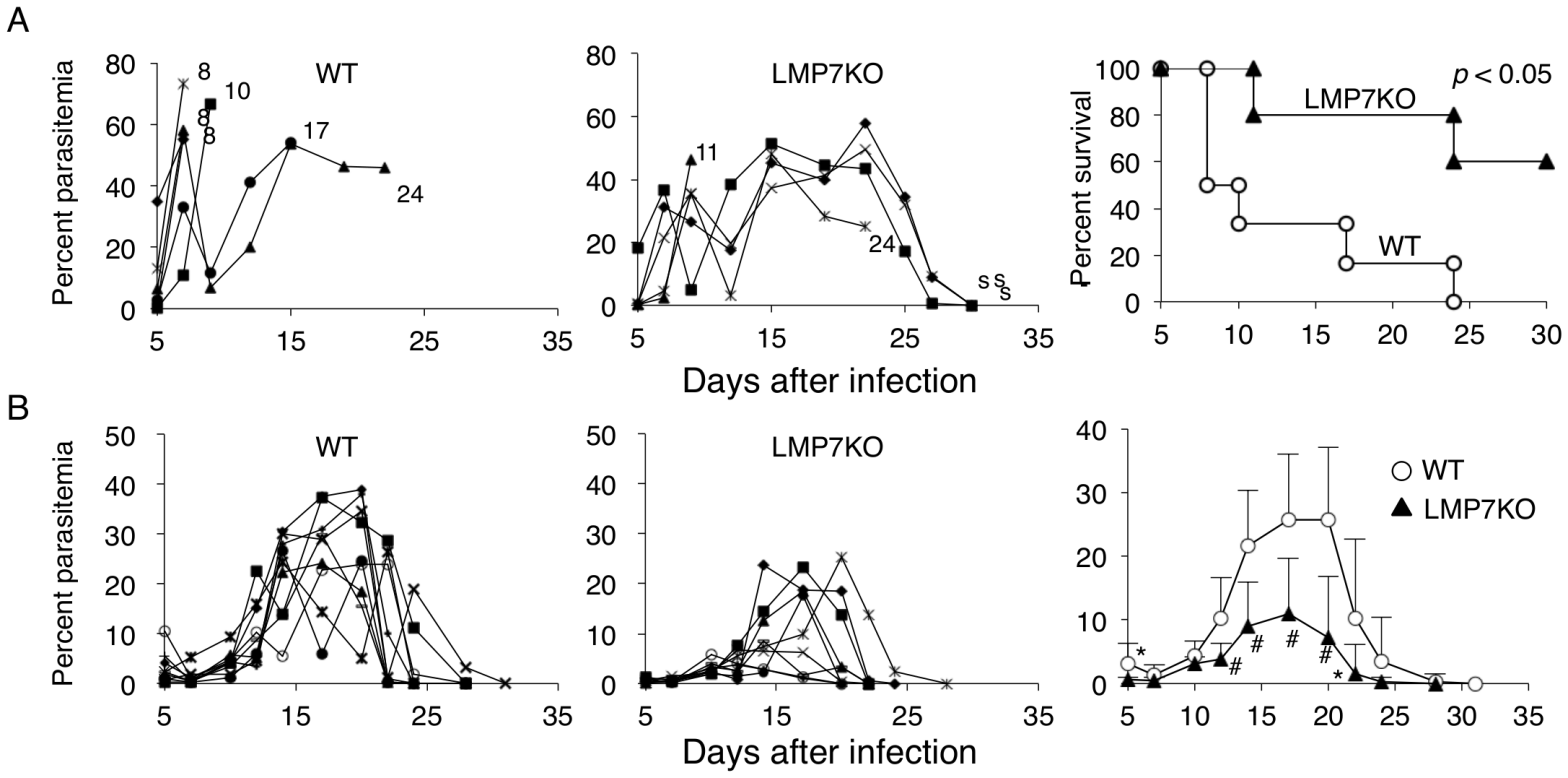
Resistance of LMP7-deficient mice to infection with *P. yoelii*. (**A**) Parasitemia (two left panels) and survival ratio (right panel) was monitored in the indicated mice infected with PyL. Each symbol represents parasitemia from an individual mouse, and the numbers or “s” represent day of mouse death or mice survival, respectively. (**B**) Infection with PyNL was analyzed as in A. The right panel shows mean parasitemia+SD of seven WT (open circles) and LMP7-deficient (triangles) mice. *,# p<0.05 or p<0.01 indicate significant difference between WT and LMP7-deficient mice using the Student’s *t*-test, respectively. Similar results were obtained from three repeated experiments.

We first infected LMP7-deficient mice with rodent malaria parasites, *P. yoelii* 17XL (PyL) and *P. yoelii* 17XNL (PyNL). As previously reported, rapid growth of PyL parasites occurred in WT mice, and all mice infected with PyL succumbed to the infection within 2 weeks (Fig. 1A). Surprisingly, LMP7-deficient mice were partially resistant to the normally lethal infection. More than half of the LMP7-deficient mice tolerated the peak of parasitemia and survived (Fig. 1A). The resistance of LMP7-deficient mice was also observed when infected with PyNL. Infection with PyNL caused a transient infection with a peak parasitemia up to 30% followed by complete eradication in WT mice (Fig. 1B). LMP7-deficient mice infected with PyNL showed a significantly lower level of parasitemia during the course of infection and took a shorter time to recover from the infection (Fig. 1B).

### Resistance of LMP7-deficient Mice is not Due to Enhancement of Adaptive Immunity

**Figure 2:**
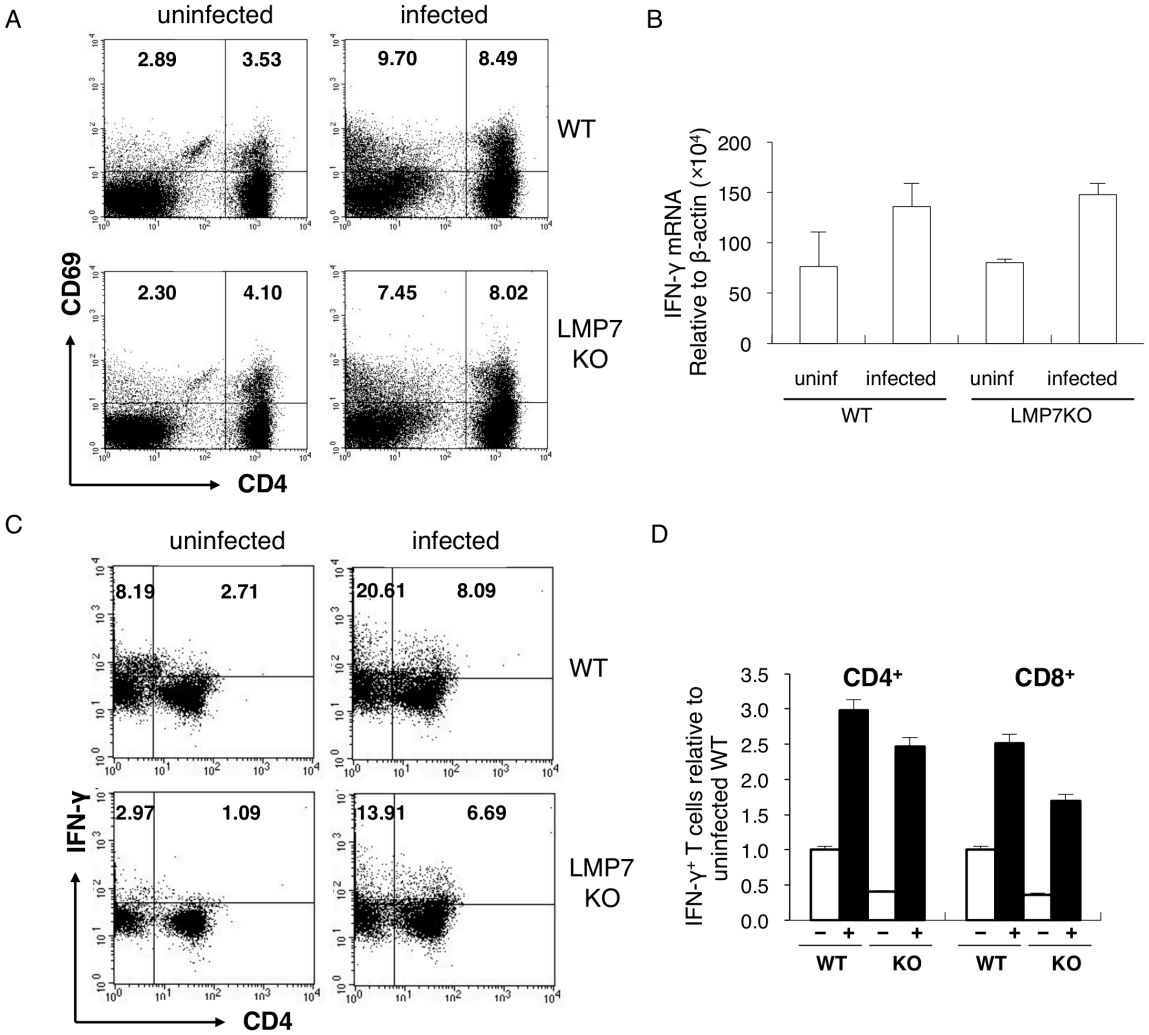
Comparable adaptive immune responses to malaria parasites in LMP7-deficient mice. Spleen cells isolated from WT and LMP7-deficient mice 5 days after infection were analyzed. (**A**) Splenocytes stained with fluorochrome-conjugated anti-CD3, anti-CD4, and anti-CD69 were analyzed for activation of T cells. Gated CD3^+^ cells were separated by CD4 and CD69 expression. The CD4^−^ cell population contained mostly CD8^+^ cells. Numbers represent the percentage of all cells in each quadrant. (**B**) mRNA encoding IFN-γ in total RNA extracted from splenocytes of the indicated mice was quantified by real-time PCR. Values represent the relative quantities of mRNA encoding genes of interest to that of β-actin and mean ± SD of 3 mice. (**C**) Production of IFN-γ in splenic T cells of the indicated mice was analyzed. Gated CD3^+^ cells were separated by CD4 and IFN-γ expression. Numbers represent the percentage of all cells in each quadrant. (**D**) Absolute numbers of IFN-γ-producing cells were also calculated (bar graph). Values indicate mean ± SD of 3 mice. Results are representative of at least two independent experiments.

LMP7-deficient mice were unexpectedly more resistant to malaria disease. To determine the mechanisms underlying resistance in LMP7-deficient mice, we analyzed immune responses in mice infected with PyL. We previously showed that LMP7-deficient mice have no defect in the number or proportions of CD4^+^ and CD8^+^ T cells in lymphoid organs compared with WT mice (26). As T cell-mediated immune responses are indispensable for protection against malaria, we first examined the activation of T cells by determining expression of CD69, an early activation marker. Splenic CD4^+^ and CD8^+^ T cells expressing CD69 were increased in response to infection with PyL in WT mice. Similarly, infection of LMP7-deficient mice activated T cells comparable to WT mice (Fig. 2A). We next analyzed the functional properties of T cells. Since IFN-γ produced by T cells is important for protection against blood-stage malaria, we examined IFN-γ production during malaria. Real-time RT-PCR analyses revealed that mRNA levels encoding IFN-γ in spleens of LMP7-deficient mice were indistinguishable from those of WT mice before and after infection with PyL (Fig. 2B). We next performed intracellular FACS analyses to confirm production of IFN-γ protein. Infection with PyL clearly increased the number of CD3^+^CD4^+^ and CD3^+^CD4^-^ (almost CD8^+^) cells that expressed IFN-γ (Fig. 2C). Although similar numbers of CD4^+^ T cells producing IFN-γ were found in LMP7-deficient mice compared to WT mice they contained less IFN-γ producing CD8^+^ T cells than WT mice (Fig. 2D). This might be caused by the less-efficient generation of antigenic epitopes for CD8^+^ T cells due to the lack of LMP7, resulting in the failure of full activation of functional CD8^+^ T cells. However, we did not observed enhanced adaptive immune responses that could explain the increased resistance to malaria observed in LMP7-deficient mice.

### Resistance of LMP7-deficient Mice is not Due to Enhancement of Innate Immunity

**Figure 3:**
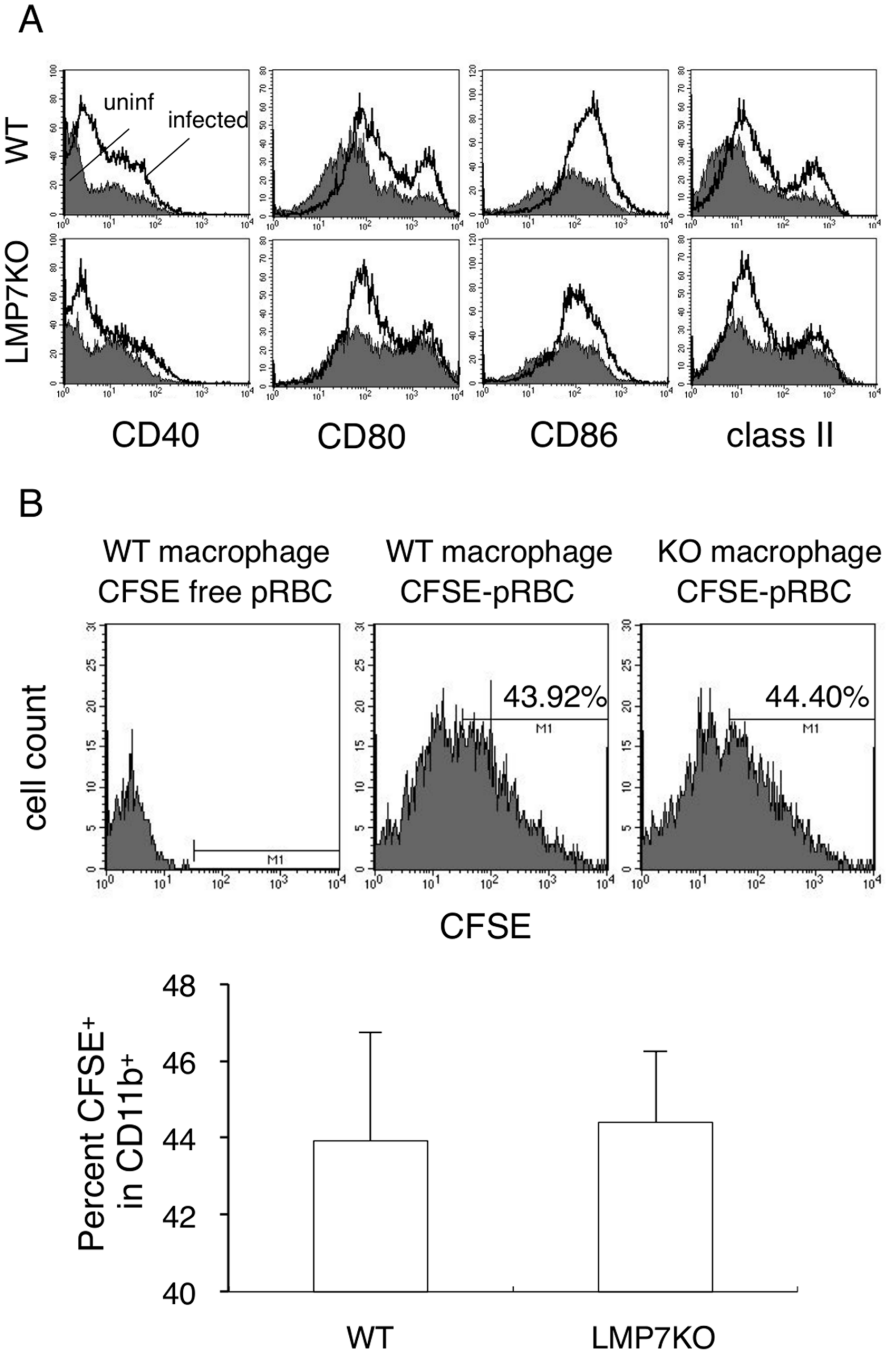
Innate immune responses against *P. yoelii* in LMP7-deficient mice. (**A**) Splenic CD11c^+^ dendritic cells obtained from WT (upper panels) and LMP7-deficient mice (lower panels) 5 days after infection were analyzed for their expression of activation markers. Histograms show expression patterns of the indicated molecules in uninfected (shaded areas) and PyL-infected mice (bold lines). (**B**) Peritoneal macrophages from WT and LMP7-deficient mice were cultured with CFSE-labeled pRBCs prepared from WT mice for 1 hour at 1∶10 ratio. After removing free RBCs by lysis with 0.83% NH_4_Cl, macrophages were stained with PE-conjugated anti-mouse CD11b antibody before flow cytometric analyses. Histograms represent CFSE intensity of gated CD11b^+^ macrophages. CFSE-positive cells were determined by fluorescence intensity of macrophages cultured with CFSE-free pRBCs (left panel). Numbers indicate percentage of CFSE-positive cells. Values in the bar graph represent mean ± SD of three mice, and statistical significance was not observed.

Since LMP7-deficient mice showed a significant lower parasitemia in the early phase of infection, which was not due to differences in adaptive immunity, we next analyzed innate immune responses exerted by DCs and macrophages. Activation of DCs critical for driving adaptive immunity occurred in both WT and LMP7-deficient mice at 5 days after infection with PyL. The degree of activation was lower in LMP7-deficient mice in terms of expression of DC activation markers (Fig. 3A). These results suggested that DCs were less activated in response to lower levels of parasites, because the level of parasitemia was significantly low at this time point in LMP7-deficient mice. Thus, activation of DCs could not explain the enhanced protection in LMP7-deficient mice.

**Figure 4:**
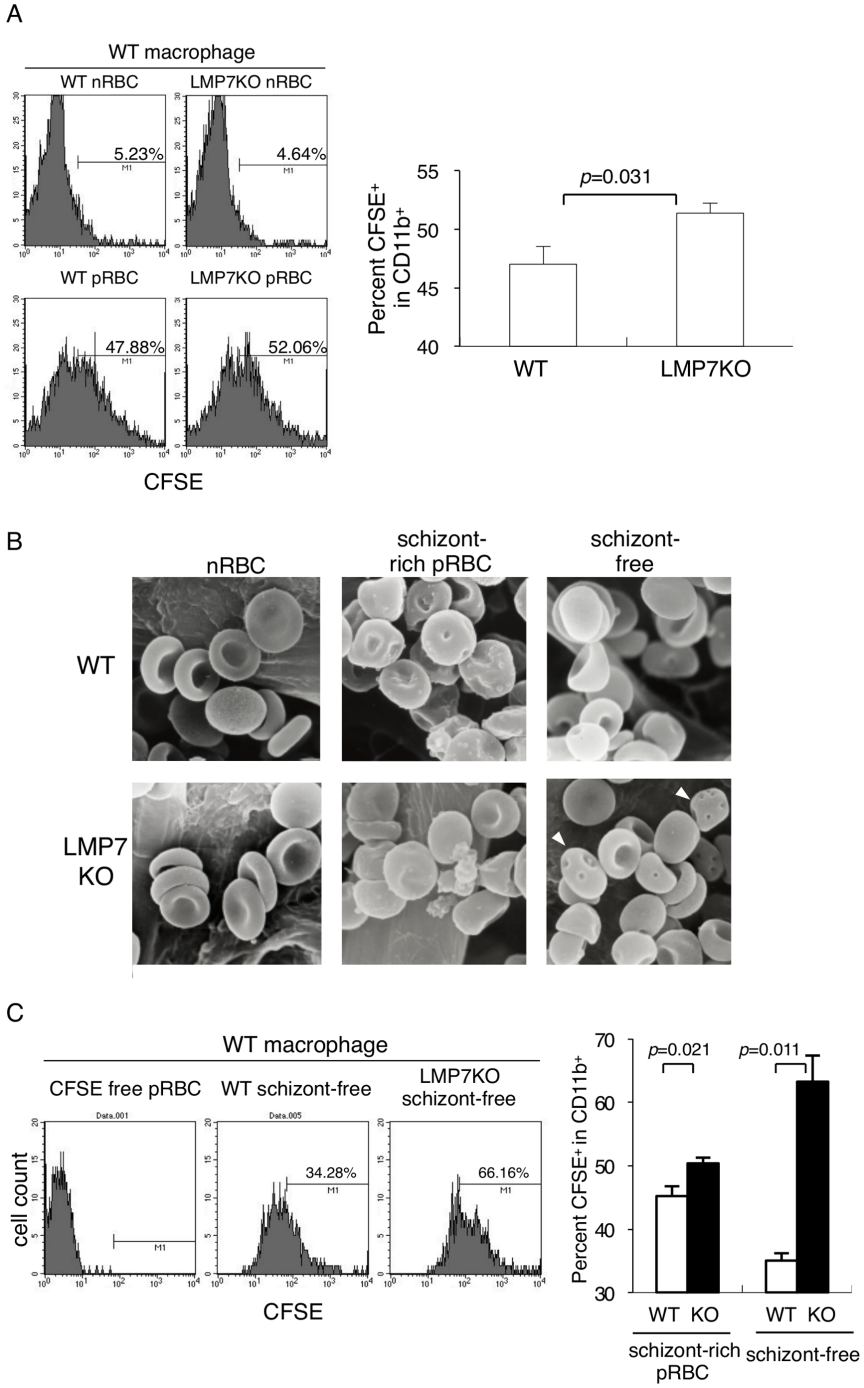
Susceptibility of RBCs from LMP7-deficient mice infected with PyL to phagocytosis by macrophages. (**A**) Peritoneal macrophages obtained from WT mice were cultured with CFSE-labeled nRBCs and pRBCs prepared from WT or LMP7-deficient mice as in Fig. 3B. Phagocytosing macrophages were determined as in Fig. 3B. Values in the bar graph represent mean ± SD of three mice, and statistical significance was evaluated by Student’s *t*-test. (**B**) Morphology of RBCs from uninfected mice (left panels), pRBCs containing late trophozoites and schizonts (center panels), and RBCs other than pRBCs (right panels) from WT (upper panels) or LMP7-deficient mice (lower panels) was examined by SEM. Arrowheads indicate deformed RBCs with small dimples. Scale bars = 10 µm. (**C**) Peritoneal macrophages obtained from WT mice were cultured with CFSE-labeled RBCs after removal of pRBCs prepared from WT or LMP7-deficient as in Fig. 3B except that the RBC to macrophage ratio was 100∶1.

Therefore, we tried to examine more primitive host defense mechanism against malaria parasites, the phagocytosis of pRBCs by macrophages. Macrophages are thought to be crucial effectors for eliminating pRBCs or free merozoites, by phagocytosis followed by their digestion in phagosomes. Schizont-rich pRBCs purified from WT mice using Percoll gradient were labeled with CFSE, cultured with macrophages, and their phagocytic ability assessed by CFSE incorporation. Phagocytosis of pRBCs by LMP-deficient macrophages was comparable to WT macrophages (Fig. 3B). Macrophages phagocytosed low numbers of RBCs from uninfected mice (Fig. 4A), suggesting that they specifically recognize some alterations in RBCs associated with infection by malaria parasites.

### RBCs of LMP7-deficient Mice are More Susceptible to Phagocytosis by Macrophages

Surprisingly, phagocytosis was remarkably enhanced when pRBCs from LMP7-deficient mice were used compared with those from WT mice (Fig. 4A), indicating that LMP7-deficient RBCs could be phagocytosed more easily by macrophages. This enhancement of phagocytosis was observed only when mice were infected with malaria parasites, as RBCs from uninfected LMP7-deficient mice were phagocytosed comparably to that from uninfected WT mice (Fig. 4A). These results suggested a difference in structure between WT and LMP7-deficient RBCs after infection. To explore the reasons why LMP7-deficient pRBCs were more sensitive to phagocytosis, we evaluated the morphology of RBCs infected with PyL using SEM. Before infection, there was no visible difference in RBCs from WT or LMP7-deficient mice, which showed a typical discoid form (Fig. 4B). Percoll gradient purified schizont-rich pRBCs composed of late trophozoites and schizonts, contained many spherical RBCs, indicating that infection with PyL altered the morphology of the RBCs. These deformations were equally observed in both WT and LMP7-deficient mice. However schizont-free RBCs, which were separated as the precipitant by Percoll gradient consisting of early trophozoites (rings) and uninfected RBCs, showed a distinct difference. RBCs from LMP7-deficient mice showed many small dimples, whereas such RBCs were rarely seen in WT mice. Quantifications based on SEM images revealed that the ratios of dimple-containing schizont-free RBCs in LMP7-deficient or WT mice were 25.33±0.19% or 4.66±2.40%, respectively (mean ± SD from 2 mice, *p* = 0.05). This morphology was not an artifact during the purification of pRBCs, because deformed RBCs were not observed in RBCs from uninfected mice processed the same way as infected mice samples.

Since schizont-free RBCs contained more deformed RBCs in LMP7-deficient mice compared with WT mice, we then analyzed phagocytosis of those RBCs by macrophages *in vitro*. As shown above, schizont-rich pRBCs from LMP7-deficient mice were phagocytosed at a greater rate than those from WT mice. Interestingly, more schizont-free RBCs from LMP7-deficient mice were phagocytosed (Fig. 4C). This remarkable difference did not reflect the proportion of ring-infected RBCs. After removal of schizont-rich pRBCs, RBC preparations from WT or LMP7-deficient mice contained 63.8% or 37.8% ring-infected RBCs, respectively. These results suggested that almost all ring-infected RBCs in LMP7-deficient were captured by macrophages, presumably resulting in the partial resistance to lethal infection with PyL in these mutant mice.

## Discussion

In this study, we analyzed the importance of LMP7 in protection against infection with malaria parasites by infecting LMP7-deficient mice with PyNL or PyL. In contrast to our expectation that the failure to activate protective CD8^+^ T cells due to the absence of LMP7 would lead to impaired protection, LMP7-deficient mice were partially resistant to malaria. Indeed, immune responses, especially CD8^+^ T cell responses, were less activated compared with WT mice. However, lack of LMP7 conferred resistance to malaria, overcoming the partially impaired activation of CD8^+^ T cells. Our results demonstrate that resistance observed in LMP7-deficient mice was attributed to the higher susceptibility of pRBCs for phagocytosis by macrophages. These results do not deny the importance of CD8^+^ T cells in protective immunity against malaria, because CD8^+^ T cells specific for LMP7-independent epitopes could be activated [37]. Indeed, substantial numbers of CD8^+^ T cells expressed CD69 and IFN-γ in response to malaria infection in LMP7-deficient mice (Fig. 2A, C). Furthermore, our previous studies clarified that the protective effects of CD8^+^ T cells are due to phagocytosis by macrophages activated by an IFN-γ dependent mechanism. In the presence of strong protection during the early phase of infection due to enhanced phagocytosis, the additional effects of CD8^+^ T cells might be difficult to observe.

Phagocytosis of pRBCs occurring in reticuloendothelial systems has been reported to be critical for the elimination of malaria parasites in various situations [38]. Attachment of pRBCs infected with *P. falciparum*, a causative agent of malignant malaria, to endothelial cells may be an escape mechanism to prevent trafficking to the spleen where phagocytosis occurs [39]. Considering these facts, it is possible that the enhanced phagocytosis is responsible for resistance of LMP7-deficient mice. As the phagocytic ability of LMP7-deficient macrophages was comparable to that of WT macrophages, the alterations of RBCs observed during infection might be important for the enhanced phagocytosis.

Under physiological conditions, macrophages in the reticuloendothelial system destroy senescent RBCs to maintain homeostasis by recognizing physical alterations in RBCs, such as lack of flexibility and deformity. Macrophages also recognize chemical and antigenic alterations. For instance, they capture “eryptotic” RBCs exposing phosphatidylserine (PS) flipped from the internal leaflet of the RBC membrane [40]. Externalization of PS is a hallmark of apoptosis in nucleated cells, which provides phagocytes with “eat-me” signals. RBCs exposing PS are also observed in iron-deficiency anemia and drug treatment enhanced phagocytosis of pRBCs during malaria, resulting in a partial protection against malaria [41], [42]. Thus, we examined the exposure of PS in RBCs from LMP7-deficient mice infected with PyL. However, infection with PyL did not cause externalization of PS to the surface of RBCs in either WT or LMP7-deficient mice (data not shown).

Instead, we found that RBCs have deformations during malaria in LMP7-deficient mice. As patients with abnormal RBCs disorders such as hereditary spherocytosis suffer from splenomegaly, a hallmark of digesting abnormal RBCs and a target for physiological therapy or splenectomy [43], abnormal RBC structures could target RBCs for phagocytosis. In our study, ring-infected RBCs and uninfected RBCs other than schizont-rich RBCs showed remarkable structural changes that were highly susceptible to phagocytosis. The uptake of ring-infected pRBCs possibly disrupt the cycle of malaria parasites. The intake of small amounts of parasite-derived molecules (stimulants for innate immunity and antigens recognized by adaptive immunity) might explain the low immune responses to malaria parasites in LMP7-deficient mice. We suggest that deformation is a major cause of the higher susceptibility of pRBCs to phagocytosis followed by resistance observed in these mutants, although we could not confirm it experimentally as alterations of RBC membrane could not be artificially reproduced. Furthermore, the difference in phagocytosis could be due to other changes in the RBCs besides deformability, such as more affinity to complement on the RBCs. In addition to the susceptibility of deformed RBCs to phagocytosis, such RBCs might be refractory to invasion of merozoites. Unfortunately, this could not be evaluated because mouse malaria parasites could not be cultured *in vitro*.

Although we have not addressed how the deficiency of LMP7 led to deformed RBCs during infection, two possibilities are postulated. First, LMP7 functions in RBCs and is involved in the development of RBCs. Lack of LMP7 during the cellular development may alter membrane structures and the distribution of components responsible for intracellular homeostasis. Thus, these resultant RBCs could not manage harmful conditions associated with malaria, such as oxidative stress [44] or physiological stress. However, previous studies have reported that RBCs only contain constitutive proteasomes, and not immune proteasomes [45], [46]. We also confirmed that LMP7 is not expressed in RBCs even after infection (data not shown). Therefore, the developmental defects, if any, must occur in erythroblasts before maturation of RBCs. Second, LMP7 functions in other cell types other than RBCs, possibly including immune cells. It has been reported that inflammatory responses induce proteins associated with cytoprotection, such as stress proteins [47]. Lack of cytoprotective effects during malaria may cause RBCs to deform. However, unfortunately the higher deformability of LMP7-deficient RBCs could not be assessed because factors during infection inducing deformation are unknown. Anyway, it would be of great interest to examine membrane-associated and cytosolic proteins of RBCs in LMP7-deficient mice. Such approaches exploring these unexpected results may reveal novel host-parasite relationships in malaria.
